# Validating Earnings in the German National Educational Panel Study - Do Interviewers Have an Impact on Measurement Accuracy?

**DOI:** 10.23889/ijpds.v1i1.308

**Published:** 2017-04-19

**Authors:** Manfred Antoni, Basha Vicari, Daniel Bela

**Affiliations:** Institute for Employment Research; Leibniz Institute for Educational Trajectories

## Objectives

We investigate characteristics of respondents and interviewers influencing the accurateness of reported income by comparing survey data with administrative data. Questions on sensitive topics like respondents' income often produce relatively high rates of item nonresponse or measurement error. In this context several analyses have been done on item nonresponse, but little is known about accuracy of reporting. Existing evidence shows that it is unpleasant for respondents to report very low or very high income. In presence of an interviewer income questions might produce incorrect responses due to social desirability bias. On the other hand side interviewers can create a trustful atmosphere in which respondents give more accurate answers.

## Method

Using linked survey and administrative data we are able to measure the extent of deviation between reported and recorded incomes and explore the influence of respondent and interviewer characteristics on it. The starting point for the linkage is data from the German National Educational Panel Study (NEPS), Starting Cohort 6, which surveys adults from birth cohorts 1944 to 1986. More than 90% of the respondents consented to a linkage of their survey information with administrative data from the German Federal Employment Agency. These longitudinal earnings data are highly reliable as they are based on mandatory notifications of employers to the social security system.

We include interviewer and respondent characteristics as well as their interactions into our model to estimate their respective impact on the incidence and size of any bias in reported incomes. This allows us to control for latent interviewer traits that might have influenced the respondent's answering behavior during each interview of a given interviewer.

## Results

The average deviation of reported from administrative earnings is relatively small (less than 10% of median earnings). Descriptive evidence shows only small variation of deviation across subgroups. Most importantly, female respondents show higher report accuracy. Multivariate results hint at a negligible influence of interviewer characteristics. The major predictors for deviation in respondents' characteristics are their sex, their absolute monthly personal income, their educational level and being born abroad.

## Conclusion

Although the average measurement accuracy is rather high, there are some differences in deviations by subgroups. The impact of these deviations depends on the research question at hand. Research with a strong focus on the respondent's earnings, e.g. when using them as a dependent variable, should use the linked data rather than only the NEPS survey data.

